# The rhizobial type III effectors ErnA and Sup3 hijack the SUMOylation pathway to trigger nodule formation in *Aeschynomene* species

**DOI:** 10.1111/nph.70334

**Published:** 2025-06-22

**Authors:** Fazal Haq, Alicia Camuel, Mélanie Carcagno, Emanuele G. Biondi, Valérie Pacquit, Laurent Deslandes, Eric Giraud, Peter Mergaert

**Affiliations:** ^1^ Institute for Integrative Biology of the Cell (I2BC) Université Paris‐Saclay, CEA, CNRS 91198 Gif‐sur‐Yvette France; ^2^ IRD, Laboratoire des Symbioses Tropicales et Méditerranéennes (LSTM) UMR IRD/Institut Agro/INRAE/Université de Montpellier/CIRAD TA‐A82/J Campus de Baillarguet 34398 Montpellier cedex 5 France; ^3^ PHIM Plant Health Institute of Montpellier Université Montpellier, IRD, CIRAD, INRAE, Institut Agro Montpellier France; ^4^ Laboratoire des Interactions Plantes‐Microbes‐Environnement (LIPME) Université de Toulouse, INRA, CNRS F‐31326 Castanet‐Tolosan France

**Keywords:** Common Symbiosis Signaling Pathway, Nod factor, nodulation, protease, rhizobia, SUMOylation, type III effector

## Abstract

- Rhizobial type III effectors (T3Es) play a crucial role in the symbiotic relationship between rhizobia and legumes by manipulating host cellular processes to promote nodule formation. Previously, we identified two T3Es, ErnA and Sup3, that trigger nodulation in *Aeschynomene* spp. in the absence of Nod factors. Here, we further investigate the mode of action of these T3Es during root nodule symbiosis.
- We employed protein interaction assays, *in vitro* binding and enzymatic activity assays, mutational analyses, and functional nodulation tests to dissect the roles of ErnA and Sup3 and their interactions with the host Small Ubiquitin‐like MOdifier (SUMO) pathway (SUMOylation).
- We demonstrate that ErnA contains a SUMO‐interacting motif (SIM) at its C terminus, which promotes its interaction with SUMO proteins *in vitro* and in plant nuclei. Additionally, we show that Sup3 possesses a C‐terminal SUMO protease domain, which not only interacts with SUMO proteins *in vitro* and in the nucleus but also exhibits SUMO protease activity. Deletion of the SIM in ErnA or mutation of the catalytic site in Sup3 abolished their ability to trigger nodulation in *Aeschynomene indica*.
- These findings suggest that type III secretion system‐dependent symbiosis is regulated by posttranslational modification through SUMOylation and that ErnA and Sup3 modulate this SUMOylation pathway to trigger nodulation.

## Introduction

The symbiosis between legumes and nitrogen‐fixing soil bacteria, known as rhizobia, is a mutualistic interaction essential for plant growth in nitrogen‐poor soils (Yang *et al*., 2022). The association results in the formation of root organs called nodules housing the bacteria that fix nitrogen to support plant nutrition. This complex process begins with flavonoid secretion by leguminous plants that induce rhizobia to produce Nod factors (NFs), key molecules in establishing the symbiotic relationship (Poole *et al*., 2018). The plant's recognition of NFs by NF receptors triggers a signaling cascade, involving the Common Symbiosis Signaling Pathway (CSSP) that also controls the symbiosis of plants with mycorrhizal fungi (Radhakrishnan *et al*., 2020). The activation of the signaling cascade leads to nodule organogenesis and its concomitant infection by the rhizobia (Crespi & Frugier, 2008; Oldroyd, 2013; Rübsam *et al*., 2023). Although NF‐based symbiosis signaling is by far the most common mechanism in legumes, some rhizobium–legume interactions do not use NFs but alternative signals to trigger nodulation. One such alternative consists of type III effectors (T3Es), which are secreted by rhizobia into root cells via the type III secretion system (T3SS) (Deakin & Broughton, 2009; Okazaki *et al*., 2013; Teulet *et al*., 2019, 2022; Ratu *et al*., 2021; Camuel *et al*., 2023).

Rhizobial T3Es, also known as nodulation outer proteins (Nops), play a significant role in the rhizobium–legume symbiosis (Deakin & Broughton, 2009; Teulet *et al*., 2022). Most of them act similarly as T3Es of pathogenic bacteria and interact with the immune system of the host plant during infection. These effectors can either promote or inhibit symbiosis depending on the host plant species (Staehelin & Krishnan, 2015; Teulet *et al*., 2022). T3Es can subvert regulators of immunity and thereby suppress plant defense responses to facilitate nodule infection and promote symbiosis. However, they can also activate effector‐triggered immunity (ETI) when recognized by plant immune receptors, in that case leading, on the contrary, to a strong immune response and halting the symbiotic interaction (Yang *et al*., 2010; Sugawara *et al*., 2018).

Unexpectedly, some rhizobial T3Es induce nodule formation and bypass the requirement for NFs. This type of T3Es is called ET‐Nods (effectors triggering nodulation) (Busset *et al*., 2021). For example, Bel2‐5 from *Bradyrhizobium elkanii* USDA61 induces nodulation of soybean in the absence of NF production or perception (Ratu *et al*., 2021). Similarly, ErnA from *Bradyrhizobium vignae* ORS3257 and Sup3 from *Bradyrhizobium* strains NAS96.2 and WSM1744 trigger nodule formation on *Aeschynomene evenia* and *A. indica*, independently of NFs (Teulet *et al*., 2019; Camuel *et al*., 2023). Strikingly, ectopic expression of ErnA or Sup3 in roots of *A. indica* and *A. evenia* results in the formation of nodule‐like structures or pseudo‐nodules, demonstrating that the activity of these effectors alone in root cells is sufficient to trigger the plant nodulation program in both species (Teulet *et al*., 2019; Camuel *et al*., 2023, 2024). Additionally, other effectors, such as NopT and NopL from *Sinorhizobium fredii* strains, have been shown to modulate nodulation either positively or negatively by interacting with NF receptors or degrading them (Bao *et al*., 2025; Ma *et al*., 2024). Together, these findings highlight an overlap between T3Es and symbiotic signaling, though the precise mode of action of the ET‐Nod T3Es remains to be elucidated.

ErnA is a novel type of T3E with no characterized functional domains but in agreement with a predicted nuclear localization signal as well as DNA‐binding domains, it was found to be targeted into the nucleus where it binds nucleic acids, suggesting its role in transcriptional programming (Teulet *et al*., 2019). Sup3 is also targeted to the nucleus and possesses a C‐terminal small ubiquitin‐like modifier (SUMO)‐protease domain, similar to the ET‐Nod Bel2‐5 or the immunity‐interfering effector NopD (Xiang *et al*., 2020; Ratu *et al*., 2021; Camuel *et al*., 2023). SUMO proteins are small proteins used by eukaryotic cells to modify specifically various substrate proteins by covalently attaching or detaching SUMO proteins. These posttranslational modifications, known as SUMOylation or deSUMOylation, can influence several aspects of the protein biology, including stability, activity, location, and interactions with protein partners (Morrell & Sadanandom, 2019). The SUMO‐tag regulates protein function by recruiting other proteins, which often involves a noncovalent interaction between SUMO and a SUMO‐interacting motif (SIM) in the interacting proteins (Morrell & Sadanandom, 2019). SIMs are small motifs consisting of a short stretch of hydrophobic residues flanked on one side or the other by negatively charged residues. SUMOylation is a cyclical and dynamic process, responding to diverse stimuli. SUMO conjugates are introduced by E3 ligases. While SUMO proteases are cysteine proteases that reverse SUMOylation by cleaving off the SUMO moiety of target proteins. In this study, we aimed to investigate the molecular mechanisms by which the ErnA and Sup3 effectors influence the initiation of nodulation in host plants. Our findings indicate that both are involved in modulating the SUMOylation pathway, which is therefore a crucial regulatory mechanism in the triggering of the nodulation signaling cascade in the absence of NFs.

## Materials and Methods

### Bacterial strains and plasmids

The bacterial strains and plasmids used in this study are listed in Supporting Information Table S1. The primers are provided in Table S2. *Escherichia coli* strains were cultured in a Luria‐Bertani (LB) medium, composed of 5 g l^−1^ yeast extract, 10 g l^−1^ NaCl, and 10 g l^−1^ tryptone or on LB agar plates, at 37°C. *Agrobacterium* strains were grown in an LB medium supplemented with rifampicin at 28°C. *Bradyrhizobium* strains were grown in a yeast mannitol (YM) medium, composed of 10 g l^−1^ mannitol, 0.5 g l^−1^ K_2_HPO_4_, 0.2 g l^−1^ MgSO_4_, 0.1 g l^−1^ NaCl, and 1 g l^−1^ yeast extract or on YM agar plates at 28°C. Where appropriate, antibiotics were used at the following concentrations (μg ml^−1^): kanamycin (50–100), rifampicin (50), ampicillin (100), and spectinomycin (100).

### Plant materials and symbiotic analysis

*Aeschynomene indica* L. was used to characterize the symbiotic phenotypes. The plants were cultivated as described previously (Okazaki *et al*., 2016). Plant inoculation and nodulation assays were performed as per the method described previously (Bonaldi *et al*., 2010). *Nicotiana benthamiana* plants were grown in a growth chamber set to 25°C with a 16 h : 8 h, light : dark photoperiod. For all the experiments, 5‐wk‐old *N. benthamiana* plants were used.

### Yeast‐two‐hybrid assays

The interactions between ErnA and Sup3 with *Aeschynomene evenia* (C. Wright) SUMO proteins were tested using yeast‐two‐hybrid (Y2H) assays. Full‐length effector genes *ernA* (of ORS3257) and *sup3* (of WSM1744) were PCR‐amplified from pVO155‐pm‐ernA::*ernA*_*3257*_ and pVO155‐pm‐*ernA*::*sup3*_1744_ plasmids, respectively (Camuel *et al*., 2023). Primers used for *ernA* amplification were ErnA‐F(SmaI) and ErnA‐R(+stop)(PstI), while *sup3* was amplified using Sup3‐F (Gib‐pGAD)(WSM1744), and Sup3‐R(+stop)(Gib‐pGAD)WSM (Table S2). The amplified products were cloned into the prey vector pGAD‐C1 (LEU2), generating pGADC1‐ErnA and pGADC1‐Sup3 constructs. Cloning of *ernA* was performed via restriction digestion using SmaI and PstI, whereas *sup3* was inserted using Gibson assembly.

Three SUMO genes from *A. evenia* accession N21/PI 225551 (gene IDs: *Ae07g02130*, *Ae07g04850*, and *Ae01g08060*) were PCR‐amplified from cDNA. The primer pairs used were Ae30‐F (SmaI)/Ae30‐R (PstI)(GG + stop), Ae50‐F (SmaI)/Ae50‐R (PstI)(GG + stop) and Ae60‐F (SmaI)/Ae60‐R (PstI)(GG + stop) (Table S2). The SUMO PCR products were cloned into the bait vector pGBDU‐C1 (URA3) using SmaI/PstI restriction digestion, resulting in pGBDUC1‐Ae30, pGBDUC1‐Ae50, and pGBDUC1‐Ae60 plasmids. Notably, the SUMO proteins expressed correspond to their processed form, ending with Gly‐Gly residues.

Y2H phenotypic assays were performed to assess protein–protein interactions as previously described (Marchadier *et al*., 2011). Briefly, the bait and prey plasmids purified from *E. coli* were introduced into the yeast strains PJ69‐4a (for bait) and PJ69‐4α (for prey) using the LiAc/PEG transformation method. Mating of the haploid yeast strains was allowed to proceed on rich medium for 5 h at 30°C, after which the resulting diploid cells were selected. Interaction phenotypes were evaluated based on the growth of diploid cells spotted on selective media lacking leucine, uracil, and histidine (SD‐LUH, supplemented with 5 mM 3‐amino‐1,2,4‐triazole (3AT)) or leucine, uracil, and adenine (SD‐LUA) for 48 h at 30°C. 3AT, an inhibitor of His3 activity, was added to SD‐LUH plates to repress growth due to basal *HIS3* expression and thus improve the specificity of detecting activated HIS3 transcription events. The presence of interaction was indicated by the ability of cells to grow under these selective conditions. Each experiment was repeated independently at least three times to confirm reproducibility.

### *Agrobacterium*‐mediated transient expression assay

*Agrobacterium tumefaciens* strain EHA105 harboring the desired constructs were cultured overnight at 28°C. The bacterial cultures were then harvested by centrifugation, washed, and resuspended in induction buffer (200 mM MES, pH 5.6, 10 mM MgCl_2_, 0.2 mM acetosyringone) to a final concentration of OD_600_ = 1.0. The *Agrobacterium* suspension in the induction buffer were incubated at room temperature for 2 h and then infiltrated into *N. benthamiana* leaves with needleless syringes for transient expression assays.

### Bimolecular fluorescence complementation assays

To assess the interaction between ErnA and Sup3 with SUMO protein using the Bimolecular fluorescence complementation (BiFC) approach, *ernA* (of ORS3257) and *sup3* (of WSM1744) genes were PCR‐amplified from pVO155‐pm‐*ernA*::*ernA*_*3257*_ and pVO155‐pm‐*ernA*::*sup3*_1744_ plasmids, respectively (Camuel *et al*., 2023). Amplification of *ernA* was carried out using the primers YC_ErnA‐F(Gib) and YC_ErnA‐R(Gib)(+stop), whereas *Sup3* was amplified using YC‐Sup3‐C‐F(Gib) and YC_Sup3‐R(Gib)(WSM). The PCR products were inserted into the pSNYCE vector, which contains a sequence coding for the C‐terminal fragment of yellow fluorescent protein (YFP), via Gibson assembly, resulting in constructs YC‐ErnA and YC‐Sup3.

The SUMO gene from *A. evenia* accession N21/PI 225551 (gene ID: *Ae07g02130*) was also PCR‐amplified from cDNA using the primer pair Ae30‐F gib‐YN/Ae30‐R gib‐YN(GG‐stop). The amplified SUMO product was cloned into the pSNYNE vector, containing a sequence coding for the N‐terminal fragment of YFP, via Gibson assembly to generate the SUMO‐YN construct. Notably, the SUMO protein used was in its processed form, ending with Gly–Gly residues. All plasmid constructs (YC‐ErnA, YC‐Sup3, and SUMO‐YN) were subsequently transferred into *A. tumefaciens* strain EHA105. *Agrobacterium* strain containing empty vector (EV‐YN) was used as a control.

BiFC assays in *N. benthamiana* were performed following a previously described protocol (Walter *et al*., 2004), with minor modifications. Buffer‐supplemented *Agrobacterium* strains containing YN and YC constructs were mixed in a 1 : 1 ratio and incubated at 25°C for 1 h. The *N. benthamiana* leaves were then infiltrated with this mixture using sterile, needleless syringes. The samples were analyzed at 48 h postinfiltration (hpi). Three hours before imaging, a nuclear stain, 4′,6‐diamidino‐2‐phenylindole (DAPI) at a concentration of 100 μg ml^−1^, was infiltrated into the leaves. Fluorescence imaging was conducted 48 hpi of the *Agrobacterium* strain using an SP8‐X confocal microscope to visualize the reconstituted YFP signal, with excitation at 514 nm and emission detected between 520 and 560 nm, indicating protein–protein interactions. The BiFC assays were repeated independently at least three times to ensure reproducibility.

### Protein expression and purification

The sequences encoding the full‐length Sup3, the C‐terminal region of Sup3 (Sup3‐C and Sup3‐C_D1456A_) the full‐length ErnA, and the SIM mutant ErnA (ErnA∆SIM) were cloned into the pET28b vector containing an N‐terminus 6× Histidine tag. To study *in vitro* interaction between ErnA and Sup3 with SUMO, the processed form of SUMO was cloned into the pGEX4T1 vector with an N‐terminal GST tag. For the enzymatic assays, a triple Hemagglutinin (3HA) sequence was fused with the C‐terminus of SUMO, following the Gly–Gly residues, and the resulting construct was cloned into the pGEX4T1 vector with an N‐terminus GST tag.

*Escherichia coli* strain BL21 (DE3) was used for recombinant protein expression. The BL21 cells harboring the respective plasmids were cultured in an LB medium containing appropriate antibiotics at 37°C until OD_600_ 0.5. Protein expression was induced by the addition of 0.5 mM isopropyl β‐d‐1‐thiogalactopyranoside, followed by incubation at 16°C for 18 h. To halt bacterial growth, cells were incubated on ice for 20 min and subsequently harvested by centrifugation at 5000 rpm for 10 min. The pellet was washed once with phosphate‐buffered saline, then resuspended in Lysis buffer (50 mM Tris–HCl, pH 8.0, 1 mM EDTA, 100 mM NaCl) supplemented with 1× protease inhibitor cocktail, and 1 mM phenylmethylsulfonyl fluoride (PMSF). The cells were sonicated for 10 min with a cycle of 10 s ON and 20 s OFF to prevent overheating, followed by centrifugation at 8000 rpm for 20 min at 4°C to remove debris.

6×His‐tagged proteins were purified using Ni‐NTA resins (Cat No. 88221; Thermo Scientific, Waltham, MA, USA), whereas GST‐fusion proteins were purified using the Pierce GST Spin Purification Kit (Cat No. 16107; Thermo Scientific) according to the manufacturers' protocols. The purified proteins were analyzed via SDS‐PAGE, subjected to Western blotting, enzyme tests, and pulldown assays.

### Ni‐NTA pulldown assay

His‐ErnA, His‐ErnA∆SIM, and His‐Sup3‐C were incubated with GST‐AeSUMO protein for 30 min at 25°C in interaction buffer (20 mM Tris–HCl, pH 7.4, 100 mM NaCl, 0.1 mM EDTA, and 0.2% Triton X‐100). Following incubation, prewashed Ni‐NTA resin was added to the reaction mixture and incubated for an additional 1 h at 4°C. The resin was washed with washing buffer (50 mM sodium phosphate, pH 8.0, 300 mM NaCl, 45 mM imidazole) to remove nonspecific binding. The bound proteins were eluted by adding 80 μl elution buffer (50 mM sodium phosphate, pH 8.0, 300 mM NaCl, 250 mM imidazole). Subsequently, 40 μl 3× protein loading buffer (B7703S; NEB, Ipswich, MA, USA) was added to the elution, and the mixture was heated at 100°C for 8 min. The purified proteins were detected using anti‐Glutathione‐*S*‐transferase antibody (G7781‐100UL; Sigma) and anti‐rabbit (A0545; Sigma) or 6×‐His antibody (37‐2900; Invitrogen) and anti‐Mouse antibody (A9917‐1ML; Sigma).

### SUMO protease activity

For the *in vitro* SUMO protease assay, purified GST‐SUMO‐3HA proteins were incubated with purified His‐tagged test proteins in an incubation buffer (50 mM Tris–HCl (pH 8.0), 0.5 mM EDTA, 5% glycerol, 50 mM NaCl) for 30 min at 25°C. The protease activity was evaluated using full‐length Sup3, Sup3‐C, which corresponds to the C‐terminal domain of Sup3 comprising 198 residues, and Sup3‐C_D1456A_, a Sup3‐C variant with an aspartic acid‐to‐alanine substitution at position 1456 in the catalytic core. The variant Sup3‐C_D1456A_ was generated using the Q5® Site‐Directed Mutagenesis Kit (NEB) and the primers indicated in Table S2. NopD served as a positive control in this assay. The reaction mixtures were then analyzed by Western blot using an anti‐GST antibody. The removal of the C‐terminal 3HA tag was confirmed by a detectable size shift of 3.3 kDa.

### SDS‐PAGE and western blotting

Protein samples were mixed with 3× loading buffer (B7703S; NEB) and boiled for 8 min. The samples were then separated on a 12% SDS‐PAGE gel and transferred onto a nitrocellulose membrane (10600007; Cytiva, Marlborough, MA, USA) for immunoblotting using specific antibodies as described above. Protein bands were detected using the SuperSignal chemiluminescent substrate (34580; Invitrogen) and visualized with the ChemiDoc Touch imaging system (Bio Rad).

### Mutagenesis and complementation

The Q5® Site‐Directed Mutagenesis Kit (NEB), the plasmids pVO155‐*pm*‐*ernA* and *pVO155‐pm‐sup3* containing the full‐length genes under their own promoter previously constructed (Camuel *et al*., 2023), and the primers indicated in Table S2 were used to design constructs encoding for a SIM‐deleted form of ErnA and a catalytic mutant of Sup3 (Sup3_D1456A_), in which the predicted catalytic aspartic acid residue D1456 was substituting with alanine. These constructs were then introduced into the ORS3257∆*ernA* and WSM1744∆*sup3* mutants previously obtained (Camuel *et al*., 2023) by single crossing over for complementation experiments.

## Results

### ErnA and Sup3 interact with SUMO in the nucleus

**Fig. 1:**
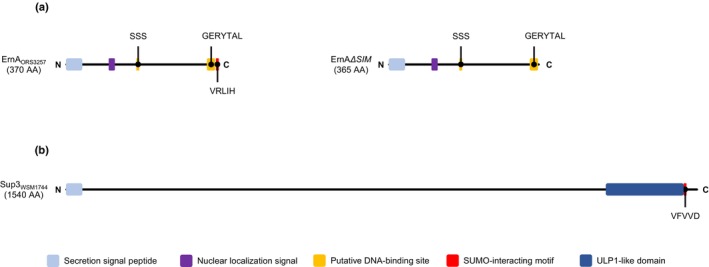
Schematic representation of ErnA_ORS3257_ and Sup3_WSM1744_ protein structure. (a) Schematic representation of ErnA from *Bradyrhizobium* strain ORS3257, highlighting the type III secretion signal peptides at the N‐terminal, nuclear localization signal (NLS), putative DNA‐binding sites, and the C‐terminal SUMO‐interacting motif (SIM). The SIM is located at the C‐terminal and is marked by a red box. This SIM was deleted to form derivative of ErnA (ErnA∆SIM). (b) Schematic representation of Sup3 from *Bradyrhizobium* strain WSM1744, highlighting at the C‐terminal ULP1‐like protease domain which also contains a SIM. SUMO, small Ubiquitin‐like mOdifiers.

ErnA does not contain any major functional domains except a type III secretion signal peptide at the N‐terminus (Teulet *et al*., 2019). However, sequence analysis using the DP‐bind and SUMO‐GPS tools identified two putative DNA‐binding sites and a C‐terminal SIM conserved in ErnA‐like proteins, respectively (Fig. 1a), suggesting ErnA targets SUMOylated host proteins in the nucleus. In addition, Sup3 possesses a C‐terminal SUMO‐protease domain (a ubiquitin‐like protease 1 (ULP1) domain) (Camuel *et al*., 2023) (Fig. 1b), again pointing to SUMOylated targets. To investigate the hypothesis that both ErnA and Sup3 have SUMOylated protein targets, we first examined their capacity to interact with SUMO proteins from *A. evenia*. This verification was particularly important for ErnA. Indeed, given the degenerate nature of the SIM consensus, it is not unlikely that some predicted SIMs do not represent true biologically relevant SUMO interaction motifs. *Aeschynomene evenia* has three canonical SUMO proteins, hereafter designated by AeSUMO30, AeSUMO50, and AeSUMO60 (gene IDs: *Ae07g02130*, *Ae07g04850*, and *Ae01g08060*) (Fig. S1a). The corresponding cDNAs of these three genes were cloned into a Y2H‐binding domain vector (bait vector), whereas the full‐length cDNAs of *ernA* (of strain *B. vignae* ORS3257) and *sup3* (of strain *B. archetypum* WSM1744) were introduced into a Y2H activating domain vector (prey vector). The binding domain vectors and activating domain vectors, including a positive control (BD‐45 and AD‐35) (Fabret *et al*., 2008) and empty vectors as a negative control, were pairwise combined with the pJ69 yeast strain, carrying the appropriate markers for selection of plasmids and growth in case of reconstitution of the GAL4 transcription factor through protein–protein interaction.

The results showed that Sup3 used as prey interacts with all three SUMO proteins from *A. evenia* expressed as bait, whereas the Y2H approach did not reveal interaction of ErnA with the tested SUMOs (Fig. S1b,c). This lack of interaction for ErnA could be attributed to several factors. First, the Y2H system requires proper folding and localization of fusion proteins, and it is possible that the ErnA fusion protein may not have folded correctly, preventing it from interacting with the SUMO proteins. Additionally, the Y2H assay relies on transcriptional activation as a readout, which can sometimes be hindered if the fusion protein interferes with the transcriptional machinery or if the bait and prey proteins are sterically hindered in the yeast nucleus (Van Criekinge & Beyaert, 1999).

**Fig. 2:**
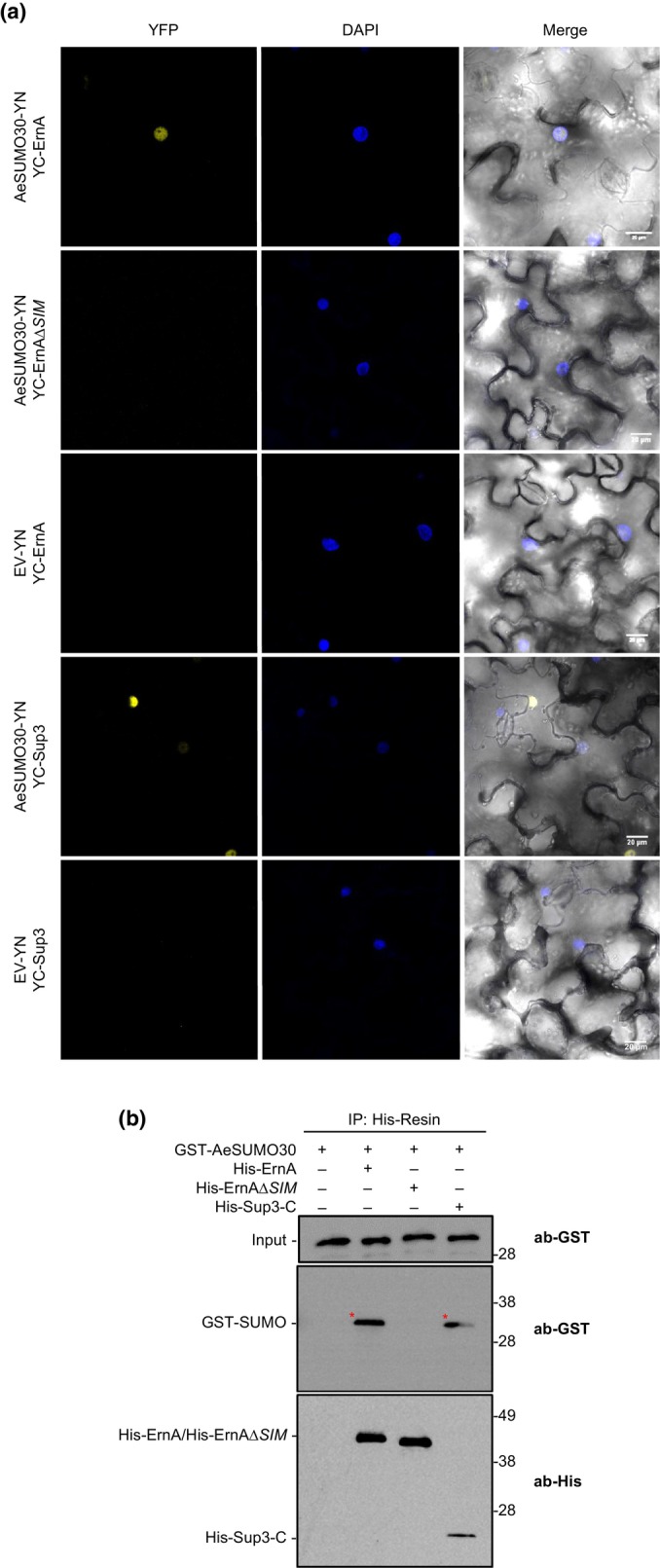
ErnA and Sup3 interact with SUMO *in planta* and *in vitro*. (a) Bimolecular fluorescence complementation (BiFC) assay showing the interaction between ErnA, Sup3, and AeSUMO30 in *Nicotiana benthamiana* nuclei. YC‐ErnA, YC‐Sup3, and AeSUMO30‐YN were co‐expressed in *N. benthamiana* leaves via *Agrobacterium*‐mediated transient expression. Controls included YC‐ErnA co‐infiltrated with empty YN vector and YC‐Sup3 co‐infiltrated with empty YN vector as well as YC‐ErnA∆SIM co‐infiltration with empty YN to show the importance of the ErnA's SIM domain in the interaction. At 48 h post‐infiltration (hpi) the leaves were visualized with confocal microscopy. DAPI staining revealed the localization of nuclei. Scale bars represent 20 μm. The experiments were repeated at least three times with similar results. (b) Pulldown assay to detect *in vitro* direct interaction between His‐tagged ErnA or His‐tagged Sup3‐C with GST‐tagged AeSUMO30 protein. The purified His‐ErnA and His‐Sup3‐C were incubated with purified GST‐SUMO protein. The co‐purified complexes were analyzed via immunoblotting using specific antibodies. The experiments were repeated at least three times with similar results. The red asterisks represent the interaction band. DAPI, 4′,6‐diamidino‐2‐phenylindole; SUMO, small Ubiquitin‐like mOdifiers; YFP, yellow fluorescent protein; SIM, SUMO‐interacting motif.

Given these potential limitations, we employed the BiFC assay in *N. benthamiana* leaves as an alternative approach to further investigate the potential interaction of ErnA with SUMOs and independently confirm the physical interaction detected between Sup3 and SUMOs in yeast. For this assay, we randomly selected AeSUMO30 (gene ID: *Ae07g02130*). We first assessed the subcellular localization of AeSUMO30 and also re‐analyzed the localization of ErnA and Sup3. The ErnA, Sup3, and AeSUMO30 proteins, each C‐terminally fused with YFP, were transiently expressed in *N. benthamiana* via *Agrobacterium*. Leaf samples were observed using confocal microscopy at 48 hpi, and results confirmed the previously reported nuclear localization of ErnA (Teulet *et al*., 2019) and Sup3 (Camuel *et al*., 2023), while AeSUMO30 protein was localized both in the nucleus and in the cytoplasm (Fig. S2). Subsequently, to investigate their ability to interact with AeSUMO30, the full‐length of *ernA*, the SIM deleted *ernA* form (*ernA*∆SIM) and the full length of *sup3* were cloned into the pSPYCE vector downstream of the sequence encoding the cYFP moiety, resulting in YC‐ErnA, YC‐ErnA∆SIM, and YC‐Sup3, respectively. The full‐length cDNA of AeSUMO30 was cloned into the pSPYNE vector upstream of the sequence coding for the nYFP moiety, resulting in AeSUMO30‐YN. YC‐ErnA and YC‐Sup3 were independently co‐expressed with SUMO‐YN in *N. benthamiana* leaves via transient *Agrobacterium*‐mediated expression. Co‐expression of YC‐ErnA and YC‐Sup3 with the empty vector served as a negative control. The infiltrated leaf samples were analyzed at 48 hpi using confocal microscopy, with DAPI infiltrated 3 h before analysis. The BiFC results showed that both ErnA and Sup3 interact with the AeSUMO30 protein, with the interaction detected inside the nucleus, whereas ErnA∆SIM did not show interaction with SUMO (Fig. 2a).

The ability of ErnA and Sup3 to physically interact with AeSUMO30 was further confirmed by an Ni‐NTA pull‐down assay. In this assay, purified GST‐tagged AeSUMO30 was incubated with recombinant 6His‐tagged ErnA or Sup3‐C (198 amino acids) produced in *E. coli*. Proteins were then incubated on an Ni‐NTA affinity resin, and pulled‐down GST‐tagged proteins were detected by immunoblot with anti‐GST antibodies. In these assays, both ErnA and Sup3‐C directly interact with SUMO protein *in vitro* (Fig. 2b), confirming their physical interaction. The SIM‐deleted ErnA does not show interaction with SUMO protein, suggesting that the SIM is required for the interaction of ErnA with SUMO proteins. These findings suggest that the mode of action of both ErnA and Sup3 resides in the interference with nuclear SUMOylation processes.

### Sup3 has SUMO protease activity but not ErnA

**Fig. 3:**
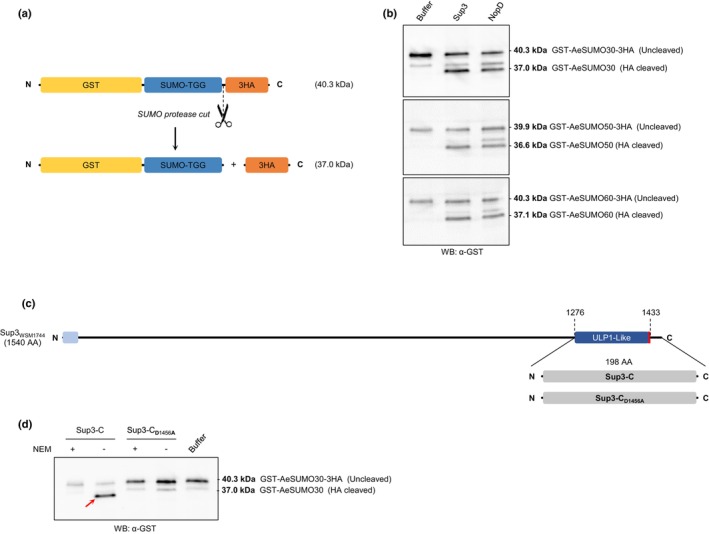
Sup3 from *Bradyrhizobium* strain WSM1744 is a functional small Ubiquitin‐like mOdifiers (SUMO) protease. (a) Schematic representation of the SUMO protease cleavage assay. GST‐SUMO‐3HA fusion proteins were used as substrates to assess protease activity. The SUMO isoforms used in this assay are from *Aeschynomene evenia*. SUMO protease cleavage results in the removal of the 3HA tag, producing a smaller GST‐SUMO protein that can be detected by western blot using an anti‐GST antibody. Reaction mixtures were incubated at 25°C for 1 h. (b) SUMO protease activity by western blot analysis. Purified GST‐AeSUMO30‐3HA, GST‐AeSUMO50‐3HA, and GST‐AeSUMO60‐3HA proteins were incubated with buffer (negative control), Sup3, or NopD (positive control, from *Bradyrhizobium* strain XS1150 2020) at 25°C for 1 h. The reaction mixtures were analyzed via immunoblotting using specific antibodies. Incubation with both Sup3 and NopD resulted in band shifts indicating the cleavage of the 3HA tag from all three SUMO isoforms. (c) Schematic of Sup3‐C (C‐terminal SUMO protease domain) and its catalytic mutant Sup3‐C_D1456A_. The catalytic aspartic acid residue in Sup3‐C was substituted with alanine to create the mutant form. (d) Protease activity of the C‐terminal domain of Sup3. GST‐AeSUMO30‐3HA was incubated with either Sup3‐C or the catalytic mutant Sup3‐C_D1456A_ at 25°C for 1 h. Western blot analysis of the reaction mixtures were performed with specific antibodies. All the experiments were performed at least three times and led to similar results. The red arrow indicates the HA cleaved GST‐AeSUMO30 band.

Sup3 is a large protein, consisting of 1463 amino acids, that shares sequence similarity with other rhizobial effectors, such as NopD and Bel2‐5, as well as with the *Xanthomonas euvesiactoria* effector XopD. Notably, Sup3 contains a C‐terminal domain that belongs to the C48 cysteine peptidase family, a ubiquitin‐like protease 1 (ULP1) domain, which suggests that it may function as a SUMO protease. To investigate whether Sup3 indeed possesses SUMO protease activity, the full‐length *sup3* gene from the *Bradyrhizobium* strain WSM1744 along with *nopD* from *Bradyrhizobium* strain XS1150, an effector with demonstrated SUMO protease activity (Xiang *et al*., 2020), were expressed as N‐terminal 6×His fusion proteins and subsequently purified. The three SUMO proteins from *A. evenia* on the other hand were expressed and purified as GST fusion proteins with an N‐terminal GST tag as well as with a C‐terminal 3HA tag positioned immediately after the terminal Gly–Gly residues, creating thereby a cleavage site for a SUMO proteases (Fig. 3a). The activity of a SUMO protease on these GST‐AeSUMO‐3HA proteins is expected to cleave off the 3HA tag, producing an *c*. 3 kDa smaller GST‐AeSUMO protein, which can be detected by western blot using an anti‐GST antibody. In agreement with a previous report (Xiang *et al*., 2020), NopD cleaved the 3HA tag from all three AeSUMO proteins (Fig. 3b). Similar to NopD, we found that Sup3 also cleaved the 3HA tag from the GST‐SUMO‐3HA proteins (Fig. 3b). Furthermore, the addition of N‐ethylmaleimide (NEM) (5 mM), a cysteine protease inhibitor (Hotson *et al*., 2003), blocked the release by both NopD and Sup3 of the low molecular weight form of the GST‐SUMO fusion protein (Fig. S3a), confirming that the observed cleavage is mediated by cysteine protease activity.

To further confirm the SUMO protease activity of Sup3, we expressed the C‐terminal SUMO protease domain of Sup3 (Sup3‐C; 198 amino acids) and its corresponding catalytic mutant by substituting the predicted catalytic aspartic acid residue D1456 with alanine (hereafter designated by Sup3‐C_D1456A_) (Fig. 3c). Both truncated proteins were N‐terminally fused with a 6×His epitope tag. As shown in Fig. 3(d), Sup3‐C, but not Sup3‐C_D1456A_, cleaved the 3HA tag from GST‐AeSUMO30‐3HA, indicating that the cleavage is dependent on its catalytic site. These results collectively demonstrate that Sup3 possesses SUMO protease activity.

In contrast to Sup3, ErnA lacks a ULP1‐like domain, suggesting that its mode of action is different from Sup3 and independent of deSUMOylation. To address this, we tested the SUMO protease activity of ErnA using the same GST‐AeSUMO‐3HA fusion proteins as substrates. Unlike Sup3, ErnA did not cleave the 3HA tag from the SUMO fusion proteins (Fig. S3b), confirming that ErnA does not possess SUMO protease activity.

### SIM of ErnA and SUMO‐protease activity of Sup3 are required for triggering nodulation in *A. indica*

**Fig. 4:**
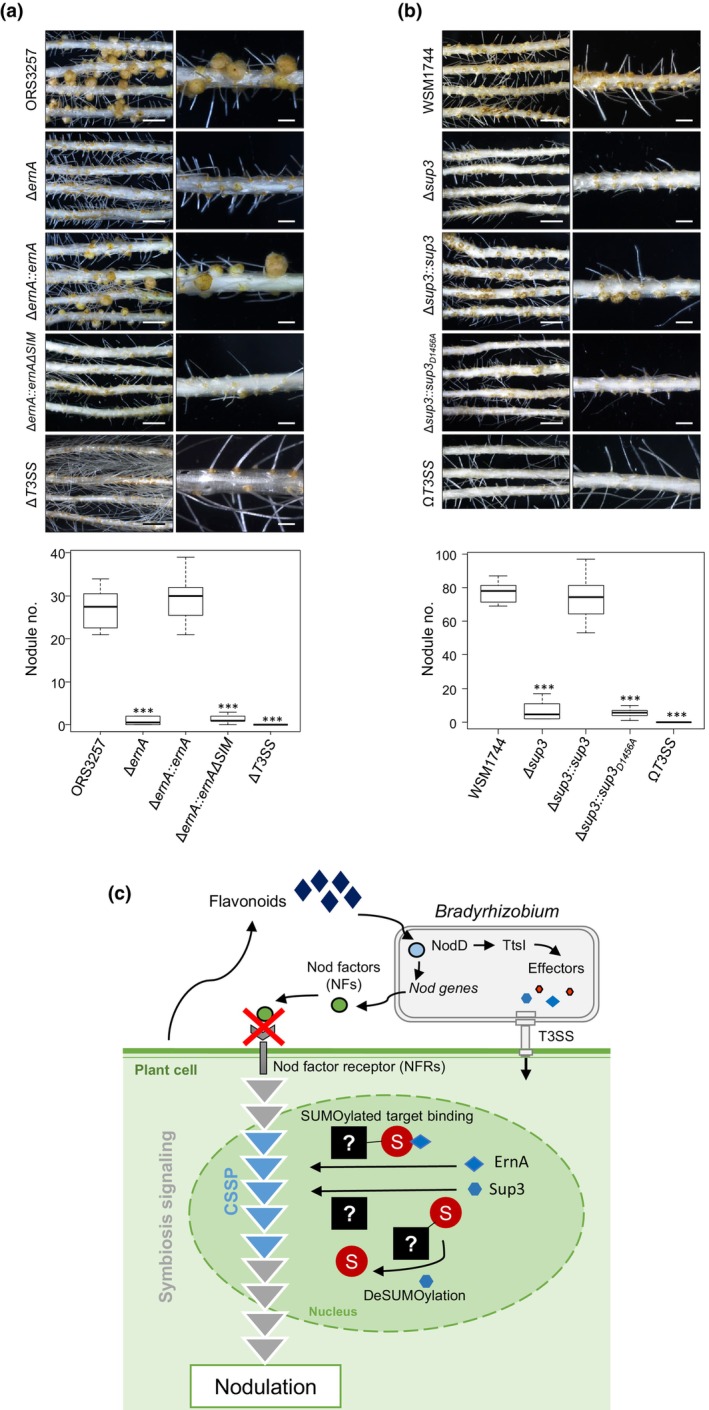
SIM‐deleted ErnA and catalytically inactive Sup3 cannot complement nodulation of ET‐Nod mutant *Bradyrhizobium* strains in *Aeschynomene indica*. (a, b) Symbiotic phenotypes of the wild‐type *Bradyrhizobium* strains ORS3257 (a) and WSM1744 (b) and their respective mutant derivatives, assessed on *A. indica*. Nodules formed by each treatment group were counted at 21 d postinoculation (dpi). Bars: (left) 0.5 cm; (right) 0.2 cm. Box plots (below) show the results of one of the three experiments independently performed on 8 plants each. The central rectangle extends from the first quartile to the third quartile; the line inside the rectangle represents the median, and the whiskers above and below the box indicate the positions of the maximum and minimum values, respectively. Significant differences compared to the wild‐type (WT) strain were assessed using a nonparametric Kruskal–Wallis test. ***, *P* < 0.001. In (a), the mutants ORS3257∆*T3SS*, ORS3257∆*ernA*, and ORS3257∆*ernA*::*ernA* were previously obtained (Camuel *et al*., 2023). The complemented mutant ORS3257∆*ernA*::*ernA∆SIM* was constructed specifically for this study. In (b), the mutants WSM1744Ω*T3SS*, WSM1744∆*sup3*, and WSM1744∆*sup3::sup3* were previously obtained (Camuel *et al*., 2023). The complemented mutant WSM1744∆*sup3::sup3*_*D1456A*_ was constructed specifically for this study. (c) Proposed model for the function of ErnA and Sup3 in modulating the SUMOylation pathway within the Common Symbiosis Signaling Pathway (CSSP) to trigger nodulation in *Aeschynomene* spp. Flavonoids released by legume roots induce the production of NFs and activate the type III secretion system (T3SS) in rhizobia via NodD and TtsI transcription factors. In canonical signaling, NFs are recognized by NF receptors, triggering a signaling cascade that activates the CSSP, essential for nodulation. In *Aeschynomene* spp., NF receptors are lacking, and independently of NFs, the ET‐Nod T3Es, like ErnA and Sup3, interfere with the SUMOylation pathway that may modulate the activity of the CSSP. ErnA, with its SUMO‐interacting motif (SIM), likely modulates the activity of SUMOylated proteins by interacting with them, while Sup3, with its SUMO protease activity, alters the SUMOylation status of key proteins. These interactions are crucial for regulating the CSSP and initiating nodulation in an NF‐independent, T3SS‐dependent manner. SUMO, small Ubiquitin‐like mOdifiers.

To determine whether the SIM is required for ErnA's function in symbiosis, we cloned *ernA* and the SIM‐deleted form of *ernA* with its native promoter into a nonreplicative vector in *Bradyrhizobium* and introduced it into the nodulation‐deficient mutant ORS3257∆*ernA* by single‐crossing over. *Aeschynomene indica* plants were inoculated with ORS3257∆*ernA*::*ernA* and ORS3257∆*ernA*::*ernA*∆SIM. Our results showed that the expression of wild‐type ErnA in ORS3257∆*ernA* restored nodulation as previously observed (Teulet *et al*., 2019), whereas ErnA∆SIM did not restored the nodulation ability of ORS3257∆*ernA* (Fig. 4a). These findings suggest that the SIM of ErnA is essential for triggering nodulation.

Similarly, to determine whether the SUMO protease activity of Sup3 is necessary for its function as an ET‐Nod, we conducted a complementation assay using the nodulation‐deficient mutant WSM1744∆*sup3* (Camuel *et al*., 2023). Both the wild‐type Sup3 and a catalytically inactive mutant, Sup3_D1456A_, were expressed in WSM1744∆*sup3* with a similar strategy as above for ErnA in strain ORS3257. The wild‐type Sup3 successfully restored nodulation capability in WSM1744∆*sup3* when inoculated on *A. indica*, whereas the Sup3_D1456A_ mutant failed to do so (Fig. 4b). These results clearly indicate that the SUMO protease activity of Sup3 is essential for its role in nodule organogenesis. Overall, these results indicated that the mode of action of both ErnA and Sup3 resides in the interference with host nuclear SUMOylation processes.

## Discussion

Rhizobial T3Es are increasingly recognized for their pivotal role in the symbiotic relationship between rhizobia and legumes (Teulet *et al*., 2022). These bacterial effectors are injected via the T3SS into plant cells, where they interfere either with the host innate immune system or with the symbiosis signaling pathway to promote successful infection and symbiotic nodule formation. In this study, we have focused on the functional role of two rhizobial ET‐Nod effectors, ErnA and Sup3, that trigger nodulation on *Aeschynomene* plants, and in particular on their interaction with the host SUMOylation pathway. The results presented here build upon existing knowledge of T3E function and provide new insights into the molecular mechanisms by which rhizobial ET‐Nods manipulate plant processes to activate the nodulation program.

We have shown that ErnA possesses a functional SIM at the C‐terminus, which is crucial for its nodulation triggering activity as an ET‐Nod. SIM mediate protein–protein interactions with SUMOylated proteins by recognizing specific SUMO isoforms through hydrophobic residues that bind to a shallow groove on SUMO's surface (Yau *et al*., 2021). The interaction between SUMO and SIM provides a crucial regulatory mechanism for controlling SUMO‐mediated cellular processes (Conti *et al*., 2014). By interacting with some SUMOylated proteins, proteins with SIMs can alter the biological function of these proteins, such as their stability, their localization, their enzymatic activity, or their interaction with various partners. For example, the SIM in the jasmonic acid receptor and ubiquitin ligase COL1 plays a critical role in transcriptional programming by regulating the stability of the transcriptional repressor JAZ6 in the jasmonic acid signaling pathway. SUMOylation of JAZ6 and binding to the SIM of COL1 interferes with its ubiquitination by COL1 and its subsequent degradation by the proteasome, thereby blocking the transcriptional activation of defense genes (Srivastava *et al*., 2018). The essential role of ErnA's SIM suggests that it enables ErnA to interact with SUMOylated host proteins to trigger nodule formation. Our previous study (Teulet *et al*., 2019) demonstrated that ErnA binds nucleic acids in the host nucleus, suggesting a role in manipulating host gene expression. Our findings here suggest that this activity involves interaction with nuclear SUMOylated proteins and may occur at a posttranslational level for regulation of still unknown protein target(s).

We further have shown that the C‐terminal domain of Sup3 has SUMO protease activity as predicted from homology and that the catalytic activity of the protease is crucial for the effector to trigger nodulation in *A. indica*. However, our experiments only confirmed the ability of Sup3 to process SUMO precursors, indicating peptidase activity, and did not assess its isopeptidase activity toward SUMO‐conjugated protein substrates. Future experiments will be needed to determine whether Sup3 can remove SUMO from modified targets, a key step to fully characterize its ULP domain functionality. Notably, canonical ULPs generally possess both activities (Mukhopadhyay & Dasso, 2007). As ErnA, Sup3 is targeted to the nucleus, suggesting that also Sup3 manipulates directly or indirectly the host cell's transcriptional machinery, likely to modulate gene expression activating the nodulation program. The involvement of microbial effectors in modulating the SUMOylome in the nucleus of host cells has been demonstrated in other plant–pathogen and symbiotic interactions. For instance, the T3E effector XopD from *Xanthomonas compestris* pv *vesicatoria* has been shown to possess SUMO protease activity, targeting SUMOylated transcription factors to suppress host defense mechanisms (Hotson *et al*., 2003). NopD, a T3E from *Bradyrhizobium* sp. XS1150, processes and cleaves SUMO‐conjugated proteins, with its protease activity dependent on a functional catalytic core. This activity is require for ETI and impairs nodulation in *Tephrosia vogelii* (Xiang *et al*., 2020). Similarly, the ET‐Nod Bel2‐5 from *Bradyrhizobium elkanii* USDA61 is another nuclear SUMO protease effector that was proposed to manipulate the host transcriptional program, to trigger nodulation in soybean in this case (Ratu *et al*., 2021).

We show here *in vitro* or *in planta* interactions between ErnA or Sup3 with SUMO proteins that do not make distinctions between the SUMO carrying protein nor the SUMO isoform. However, it is expected that in the proper cellular environment, ErnA and Sup3 display a degree of specificity, both at the level of the SUMO modified proteins and the SUMO isoform they bind and process. Although the basis for selectivity of SIM‐containing proteins is not fully understood, it is thought that SIM‐containing proteins do not solely contact the SUMO moiety. Instead, they also interact with other binding surfaces in the SUMOylated protein, which could function as determinants of specificity by mediating the formation of a cooperative complex that requires the SUMO/SIM interaction as well as additional protein–protein interactions (Yau *et al*., 2021). SUMO protease on the other hand display specificity for SUMO isoforms but are known in many cases to deSUMOylated many different substrates (Hickey *et al*., 2012; Morrell & Sadanandom, 2019).

What then can be the targets of ErnA and Sup3 in *Aeschynomene* root cells to trigger nodule organogenesis? Several *Aeschynomene* spp. use a unique symbiotic process that is independent of NFs. Although these species use an NF‐independent symbiotic process, they recruit some determinants of the CSSP pathway that have been identified in other legume species that strictly depend on rhizobial NFs and their receptors (Quilbé *et al*., 2021). Notably, genes encoding the nuclear envelope ion channel POLLUX, the nuclear kinase CCaMK, and the transcriptional regulator CYCLOPS of the CSSP, as well as the transcription factors NSP2 and NIN that are positioned immediately downstream of the CSSP are required for *Aeschynomene* nodulation. *Bradyrhizobium* strains that use ErnA or Sup3 to trigger nodulation in *A. evenia* require these CSSP genes and the downstream transcription factors, suggesting that these ET‐Nods activate directly or indirectly the CSSP (Camuel *et al*., 2024). Moreover, the nodule‐like structures formed by the ectopic expression of ErnA or Sup3 in transgenic roots of *A. indica* or *A. evenia* (Teulet *et al*., 2019; Camuel *et al*., 2023, 2024) are reminiscent of the nodule‐like structures or ‘spontaneous nodules’ induced in other legumes when gain‐of‐function mutants of CSSP components or NIN are expressed (Vernié *et al*., 2015; Quilbé *et al*., 2021). Together with our observations, these findings suggest that ErnA and Sup3 activate the nodulation program in *Aeschynomene* by interacting with one or several components of the CSSP or with an upstream regulator of it/them and that this component is regulated by SUMOylation (Fig. 4c). Thus, we propose, based on this study, that SUMOylation is a regulatory mechanism of the T3SS‐triggered nodulation/symbiosis signaling pathway.

The existence of T3Es, like ErnA and Sup3, which can trigger nodulation independently of NFs, raises intriguing questions about the evolution of legume–rhizobia symbiosis. Some authors have suggested that NF‐independent symbiosis might represent an ancestral form of symbiotic interaction, with NF‐dependent mechanisms evolving later to enhance specificity and efficiency (Bonaldi *et al*., 2010; Madsen *et al*., 2010; Okazaki *et al*., 2013). However, no conclusive evidence for this proposition exists yet, and the alternative hypothesis that the NF‐independent nodulation in *Aeschynomene* is a derived trait remains equally possible. On the other hand, the ET–Nods possibly have evolved from rhizobial T3Es that interfere with immunity signaling to suppress host defenses during nodulation. Over evolutionary time, such effectors may have been repurposed to manipulate the symbiosis‐signaling pathway. Moreover, the *Bradyrhizobium* strains carrying ET–Nods possess also the *nod* genes involved in NF synthesis that share a common evolutionary history with the T3SS gene clusters (Teulet *et al*., 2020). This observation suggests that the activation of the plant symbiotic program by these T3Es was likely, at its origin, interlinked with the canonical NF‐mediated symbiotic program. Moreover, recent genomic studies in the *Bradyrhizobium* genus have revealed the existence of atypical and ancestral T3SS gene clusters (Teulet *et al*., 2020; Ling *et al*., 2025), opening new perspectives for studying the evolution of T3Es within this bacterial genus and how T3E‐mediated nodulation may have been acquired by symbiotic strains. Further analysis of the functional domain similarities between these T3Es and those characterized in pathogenic studies may provide new insights into their evolutionary history.

In conclusion, our study demonstrates that both ErnA and Sup3 may interfere with the host SUMOylation pathway during nodule organogenesis. Our data show that ErnA SIM mutants and Sup3 catalytic mutants, which are impaired in their ability to interact with SUMO proteins or exhibit SUMO protease activity, are also deficient in triggering nodule organogenesis. These findings indicate that the mode of action of ErnA and Sup3 likely involves interference with nuclear SUMOylated protein(s) or the host SUMOylation machinery. ErnA, through its SIM motif, likely modulates host SUMOylation processes by interacting with SUMOylated proteins, while Sup3, with its SUMO protease activity, directly alters the SUMOylation status of key host proteins. These findings highlight the sophisticated strategies employed by rhizobia to control host cellular processes and establish successful symbiotic relationships. Moreover, they pave the way for future investigations aiming the identification of the SUMOylated regulators of nodulation in legumes and the deciphering of the control exerted by the SUMO modification on these regulators.

## Competing interests

None declared.

## Author contributions

FH, EG and PM conceived and designed the research. FH and AC performed the experiments and analyzed the results. MC, EGB, VP and LD provided technical assistance and help in the experiments. FH, EG and PM wrote the manuscript. All authors read, commented on, and approved the manuscript.

## Disclaimer

The New Phytologist Foundation remains neutral with regard to jurisdictional claims in maps and in any institutional affiliations.

## Supporting information


**Fig. S1** Yeast two‐hybrid (Y2H) assay testing the interaction between ErnA, Sup3, and SUMO proteins from *Aeschynomene evenia*.
**Fig. S2** Subcellular localization of ErnA, Sup3, and SUMO protein in *Nicotiana benthamiana*.
**Fig. S3** SUMO protease activity of Sup3 and ErnA.
**Table S1** Bacterial strains and plasmids used in this study.
**Table S2** Primers used in this study.
Please note: Wiley is not responsible for the content or functionality of any Supporting Information supplied by the authors. Any queries (other than missing material) should be directed to the *New Phytologist* Central Office.

## Data Availability

The data supporting the findings of this study are available within the article and Figs S1–S3 and Tables S1, S2.
