# Supplementary material for: The rhizobial type III effectors ErnA and Sup3 hijack the SUMOylation pathway to trigger nodule formation in Aeschynomene species
Source: New Phytol. 2025 Jun 22;247(4):1826–36. doi: 10.1111/nph.70334 (PMC12267903; doi:10.1111/nph.70334)
Supplement: Supplementary file 1 — Fig. S1 Yeast two‐hybrid (Y2H) assay testing the interaction between ErnA, Sup3, and SUMO proteins from Aeschynomene evenia. Fig. S2 Subcellular localization of ErnA, Sup3, and SUMO protein in Nicotiana benthamiana. Fig. S3 SUMO protease activity of Sup3 and ErnA. Table S1 Bacterial strains and plasmids used in this study. Table S2 Primers used in this study. Please note: Wiley is not responsible for the content or functionality of any Supporting Information supplied by the authors. Any queries (other than missing material) should be directed to the New Phytologist Central Office. [file NPH-247-1826-s001.pdf]

New Phytologist **Supporting Information**

Article title: The rhizobial Type-III effectors ErnA and Sup3 hijack the SUMOylation pathway to trigger nodule formation in *Aeschynomene* species

Authors: Fazal Haq, Alicia Camuel, Mélanie Carcagno, Emanuele G. Biondi, Valérie Pacquit, Laurent Deslandes, Eric Giraud, Peter Mergaert

Article acceptance date: 05 June 2025

The following Supporting Information is available for this article:

Fig. S1 Yeast two-hybrid (Y2H) assay testing the interaction between ErnA, Sup3, and SUMO proteins from *Aeschynomene evenia*.

Fig. S2 Subcellular localization of ErnA, Sup3, and SUMO protein in *N. benthamiana*.

Fig. S3 SUMO Protease activity of Sup3 and ErnA

Table S1 Bacterial strains and plasmids used in this study.

Table S2 Primers used in this study.

Fig. S1 Yeast two-hybrid (Y2H) assay testing the interaction between ErnA, Sup3, and SUMO proteins from *Aeschynomene evenia*.

(a) *Aeschynomene evenia* have three canonical SUMO proteins (gene IDs: *Ae07g02130*, *Ae07g04850*, *Ae01g08060*).

(b) (c) SUMO proteins (simply differentiated with last two digit of gene id; *30*, *50*, *60*) were co-expressed with ErnA and Sup3 in yeast strain PJ6. Yeast cells were grown on selective media (CSM, -U-L, -His(+5 mM 3AT)/-Ade). In **(c)**, tenfold dilution of the bacterial suspensions (10^0 to 10^{-3}) were spotted on the selective media. Positive control (pGAD-35/pGBD-45) and negative control (empty vector) are also shown.

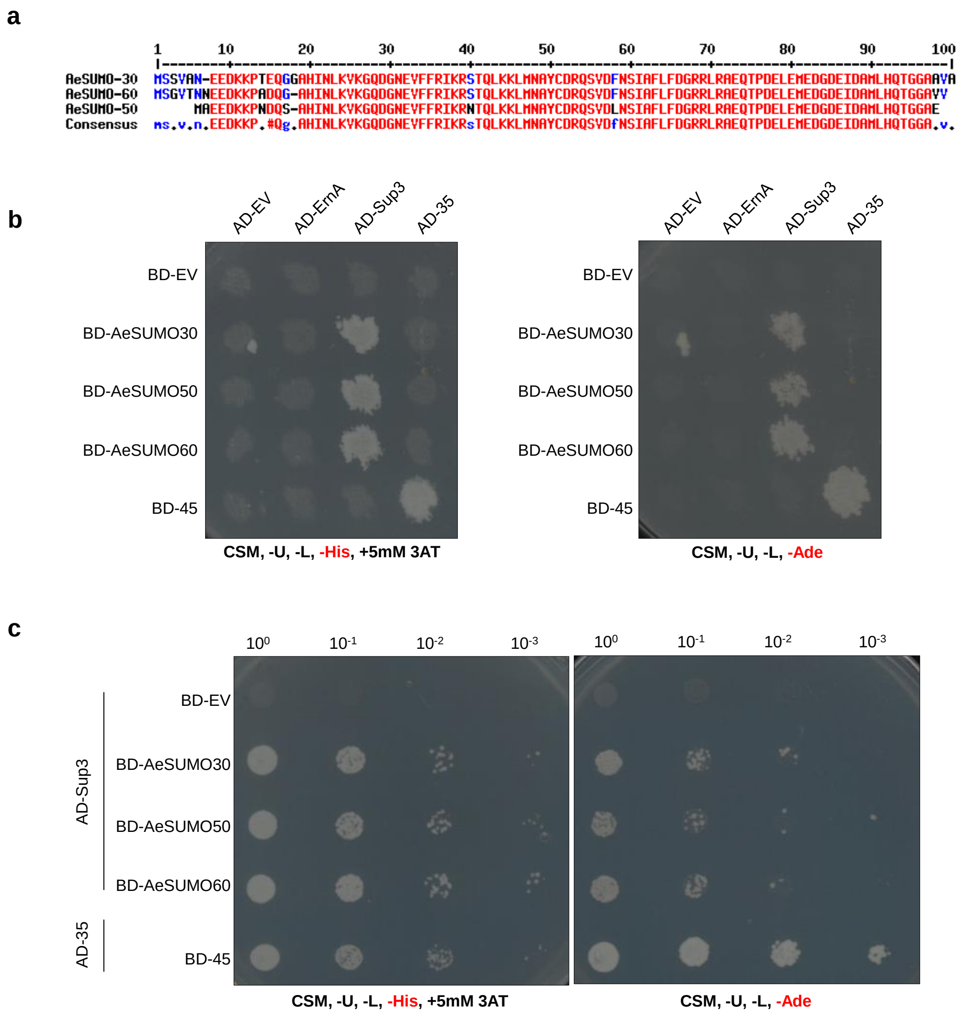

Fig. S2 Subcellular localization of ErnA, Sup3, and SUMO protein in *N. benthamiana*.

N. benthamiana leaves were inoculated with *Agrobacterium* expressing empty YFP, YFP-AeSUMO30, YFP-ErnA, YFP-ErnAΔSIM, YFP-Sup3 and YFP-Sup3$_{D1456A}$ protein. The leaf samples were visualized live at 48 hours post-infiltration (hpi) by confocal microscopy with excitation at 514 nm and emission detected between 520–560 nm. Scale bars represent 20μm. The experiments were repeated at least three times with similar results.

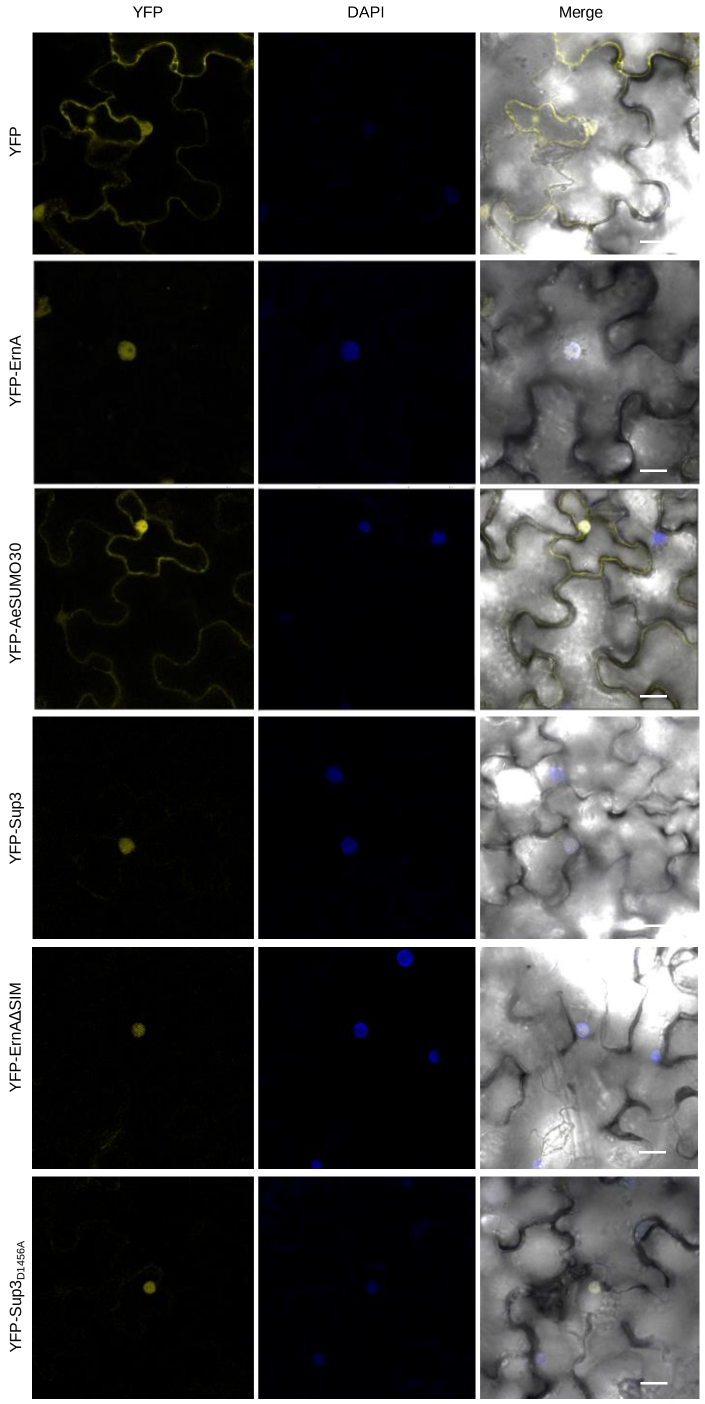

Fig. S3 SUMO Protease activity of Sup3 and ErnA

(a) Effect of N-ethylmaleimide (NEM), a cysteine protease inhibitor, on the protease activity of Sup3 and NopD. Purified GST-AeSUMO30-3HA protein was incubated with buffer (negative control), Sup3, or NopD (positive control) at 25˚C for 1 h in the presence (+) or absence (-) of 5mM NEM. The reaction mixtures were analyzed via immunoblotting using specific antibodies. The red arrow indicate the 3HA cleaved band AeSUMO30.

(b) Evaluation of ErnA SUMO protease activity. Purified GST-AeSUMO30-3HA fusion protein was incubated with buffer (negative control), ErnA, or Sup3 at 25°C for 1 h. The reaction mixtures were analyzed via immunoblotting using an anti-GST antibody. A band shift is visible in the lane corresponding to Sup3, indicating cleavage of the 3HA tag, while no band shift is observed in the lane for ErnA, reflecting the absence of SUMO protease activity.

a

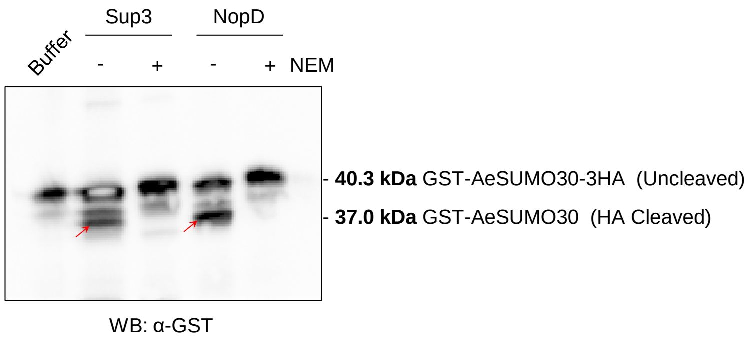

b

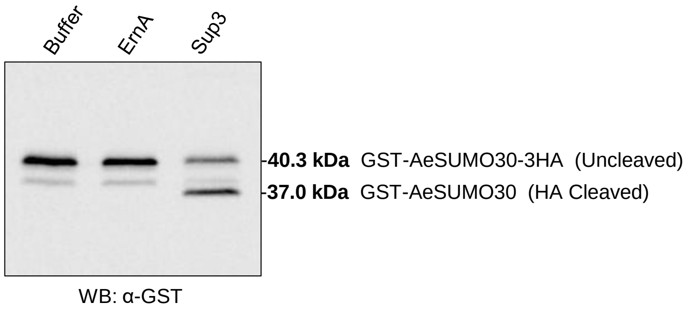

Table S1 Bacterial strains and plasmids used in this study.

Strain or plasmid	Relevant characteristics	Source
**Strain**		
Escherichia coli		
DH5α	*F-*, *endA1*, *thi-1*, *recA1*, Φ80*lacZ*, Δ M15	Invitrogen
BL21	*F-*, *ompT*, *hsdS20*, *gal*, *dcm*(DE3)	Invitrogen
Agrobacterium tumefaciens		
EHA105	C58, pTiBo542DT-DNA, Rif^r	This lab
Bradyrhizobium spp.		
WSM1744		
ORS3257		
Nas96.2		
Yeast		
PJ69-4A	*MATa trp1-901 leu2-3,112 ura3-52 his3-200 gal4Δ gal80Δ LYS2::GAL1-HIS3 GAL2-ADE2 met2::GAL7-lacZ*	(Marchadier *et al.*, 2011)
**Plasmids**		
pVO155-pm-ernA::ernA$_{3257}$	pVO155 vector containing *ernA* gene of ORS3257 and its upstream promoter region, Km^r	(Camuel *et al.*, 2023)
pVO155-pm-*ernA*::*Sup3*$_{1744}$	pVO155 vector containing *Sup3* gene of WSM1744 and upstream promoter region of *ernA*, Km^r	(Camuel *et al.*, 2023)
pGBDU-CI	used for Y2H assays, Amp^r	This Lab
pGAD-C1	used for Y2H assays, Amp^r	This Lab
pET28b(+)	pBR322 origin, *lacI*, N-terminal His-tag/thrombin/T7-Tag, an C-terminal His-Tag, Km^r	This Lab
pGBD-AeSUMO30	AeSUMO30 in *SmaI*/*PstI* site of pGBDU-C1, Amp^r	This study
pGBD-AeSUMO50	AeSUMO50 in *SmaI*/*PstI* site of pGBDU-C1, Amp^r	This study

pGBD-AeSUMO60	AeSUMO60 in *SmaI*/*PstI* site of pGBDU-C1, Amp^r	This study
pGBD-45	45 in pGBDU-C1, Amp^r	(Fabret *et al.*, 2008)
pGAD-ErnA	ErnA in *SmaI*/*PstI* site of pGAD-C1, Amp^r	This study
pGAD-Sup3	Sup3 in pGAD-C1 via Gibson cloning, Amp^r	This study
pGAD-35	35 in pGAD-C1, Amp^r	(Fabret *et al.*, 2008)
pSPYNE	Binary vector with c-myc affinity tag and C-terminus of YFP, Km^r	(Walter *et al.*, 2004)
pSPYCE	Binary vector with HA affinity tag and C-terminus of YFP, Km^r	(Walter *et al.*, 2004)
AeSUMO30-YN	AeSUMO30 in pSPYNE via Gibson cloning, Km^r	This study
YN-AeSUMO30	AeSUMO30 in pSPYNE via Gibson cloning, Km^r	This study
ErnA-YC	ErnA in pSPYCE via Gibson cloning, Km^r	This study
YC-ErnA	ErnA in pSPYCE via Gibson cloning, Km^r	This study
YC-Sup3	Sup3 in pSPYCE via Gibson cloning, Km^r	This study
pET28b(+)	pBR322 origin, *lacI*, N-terminal His-tag/thrombin/T7-Tag, an C-terminal His-Tag, Km^r	This Lab
pGEX4T1	Amp^r	
pET28b-ErnA	ErnA in with N-terminal 6xHis-tag in pET28b(+), Km^r	This study
pET28b-ErnAΔSIM	ErnAΔSIM in with N-terminal 6xHis-tag in pET28b(+), Km^r	This study
pET28b-Sup3-C	Sup3-C in with N-terminal 6xHis-tag in pGEX4T1, Km^r	This study
pET28b-Sup3-C(D_{1448}A)	Sup3-C(D_{1448}A) in with N-terminal 6xHis-tag in pGEX4T1, Km^r	This study
pGEX-AeSUMO30	AeSUMO in with N-terminal GST-tag in pGEX4T1, Amp^r	This study
pGEX-AeSUMO30-3HA	AeSUMO30-3HA with N-terminal GST-tag in pGEX4T1, Amp^r	This study
pGEX-AeSUMO50-3HA	AeSUMO50-3HA with N-terminal GST-tag in pGEX4T1, Amp^r	This study
pGEX-AeSUMO60-3HA	AeSUMO60-3HA with N-terminal GST-tag in pGEX4T1, Amp^r	This study

Abbreviations: Amp^r, ampicillin; Km^r, Kanamycin; Rif^r, Rifampicin

Table S2 Primers used in this study.

Primer name	Sequence (5’-3’; restriction sites underlined)	Description
ErnA-F (SmaI)	TCCCCCGGGATGGACCCATTTAACCGCATC	Amplifies *ernA*; contains *SmaI*/*PstI* sites
ErnA-R(+Stop)(PstI)	AACTGCAGCTATGGATGATGGATGAGCCGAAC	
Sup3-F (Gib-pGAD)WSM1744	CCAAACCCAAAAAAAGAGATCGAATTCATGAAGTTCCAATCCAC	Amplifies *sup3* for Y2H; contains upstream and dowstream DNA sequence from pGAD vector for gibson cloning
Sup3-R (+Stop)(Gib-pGAD) WSM	GACATCGATGGATCCCCCGGGTTAGCCCAAGCCG	
Ae30-F (SmaI)	TCCCCCGGGATGTCGAGTGTTGCGAACGAG	Amplifies *AeSUMO30*; contains *SmaI*/*PstI* sites
Ae30-R (PstI)(GG+stop)	AACTGCAGTCAACCTCCTGTCTGATGAAGCATC	
Ae50-F (SmaI)	TCCCCCGGGATGGCTGAGGAAGACAAGAAGC	Amplifies *AeSUMO50*; contains *SmaI*/*PstI* sites
Ae50-R (PstI)(GG+stop)	AACTGCAGTCATCCGCCAGTTTGGTGCAAC	
Ae60-F (SmaI)	TCCCCCGGGATGTCGGGCGTGACCAAC	Amplifies *AeSUMO60*; contains *SmaI*/*PstI* sites
Ae60-R (PstI)(GG+stop)	AACTGCAGTCAGCCGCCTGTCTGATGAAG	
YC_ErnA-F (Gib)	CTCTCGGCATGGACGAGCTGTACAAGATGGACCCATTTAA	Amplifies *ernA* for BiFC; contains upstream and dowstream DNA sequence from pSPYCE vector for gibson cloning
YC_ErnA-R (Gib) +stop	CCAAATGTTTGAACGATCGGGGAAATTCCTATGGATGATGGAT	
YC_Sup3-C-F Gib (WSM)	CTCTCGGCATGGACGAGCTGTACAAGATGCGCAGTGGACTA	Amplifies *sup3* for BiFC; contains upstream and dowstream DNA sequence from pSPYCE vector for gibson cloning
YC_Sup3-R Gib (WSM)	CCAAATGTTTGAACGATCGGGGAAATTCTTAGCCCAAGCC	
Ae30-F gib-YN	CTGGCGCGCCACTAGTGGATCCATGTCGAGTGTTG	Amplifies *ernA* for BiFC; contains upstream and dowstream DNA sequence from pSPYNE vector for gibson cloning
Ae30-R gib-YN(GG-stop)	ATCCCGGGAGCGGTACCCTCGAGACCTCCTGTCTGAT	
ErnASIM-stop-F	GGGGCCGGGATGACCCAATGA	Amplifies *ernAΔSIM* for complementation assays
ErnASIM-stop-R	AAGAGGGCCGTGTAACGC	
Sup3-WSM1744-site2-F	GGGGCCGGGATGACCCAATGA	Amplifies *sup3* catalytic mutant ($sup3_{D1456A}$) for complementation assays
Sup3-WSM1744-Site2-R	AAGAGGGCCGTGTAACGC	

References

Camuel A, Teulet A, Carcagno M, Haq F, Pacquit V, Gully D, Pervent M, Chaintreuil C, Fardoux J, Horta-Araujo N, *et al.* 2023. Widespread *Bradyrhizobium* distribution of diverse Type III effectors that trigger legume nodulation in the absence of Nod factor. *The ISME Journal* **17**: 1416–1429.

Fabret C, Cosnier B, Lekomtsev S, Gillet S, Hatin I, Maréchal PL, Rousset JP. **2008**. A novel mutant of the Sup35 protein of *Saccharomyces cerevisiae* defective in translation termination and in GTPase activity still supports cell viability. *BMC Molecular Biology* **9**: 22.

Marchadier E, Carballido-López R, Brinster S, Fabret C, Mervelet P, Bessières P, Noirot-Gros M-F, Fromion V, Noirot P. **2011**. An expanded protein-protein interaction network in *Bacillus subtilis* reveals a group of hubs: Exploration by an integrative approach. *Proteomics* **11**: 2981–2991.

Walter M, Chaban C, Schütze K, Batistic O, Weckermann K, Näke C, Blazevic D, Grefen C, Schumacher K, Oecking C, *et al.* 2004. Visualization of protein interactions in living plant cells using bimolecular fluorescence complementation. *The Plant Journal* **40**: 428–438.
